# Supplementary material for: monaLisa: an R/Bioconductor package for identifying regulatory motifs
Source: Bioinformatics. 2022 Feb 23;38(9):2624–5. doi: 10.1093/bioinformatics/btac102 (PMC9048699; doi:10.1093/bioinformatics/btac102)
Supplement: btac102_Supplementary_Data [file btac102_supplementary_data.pdf]

Supplementary Figures

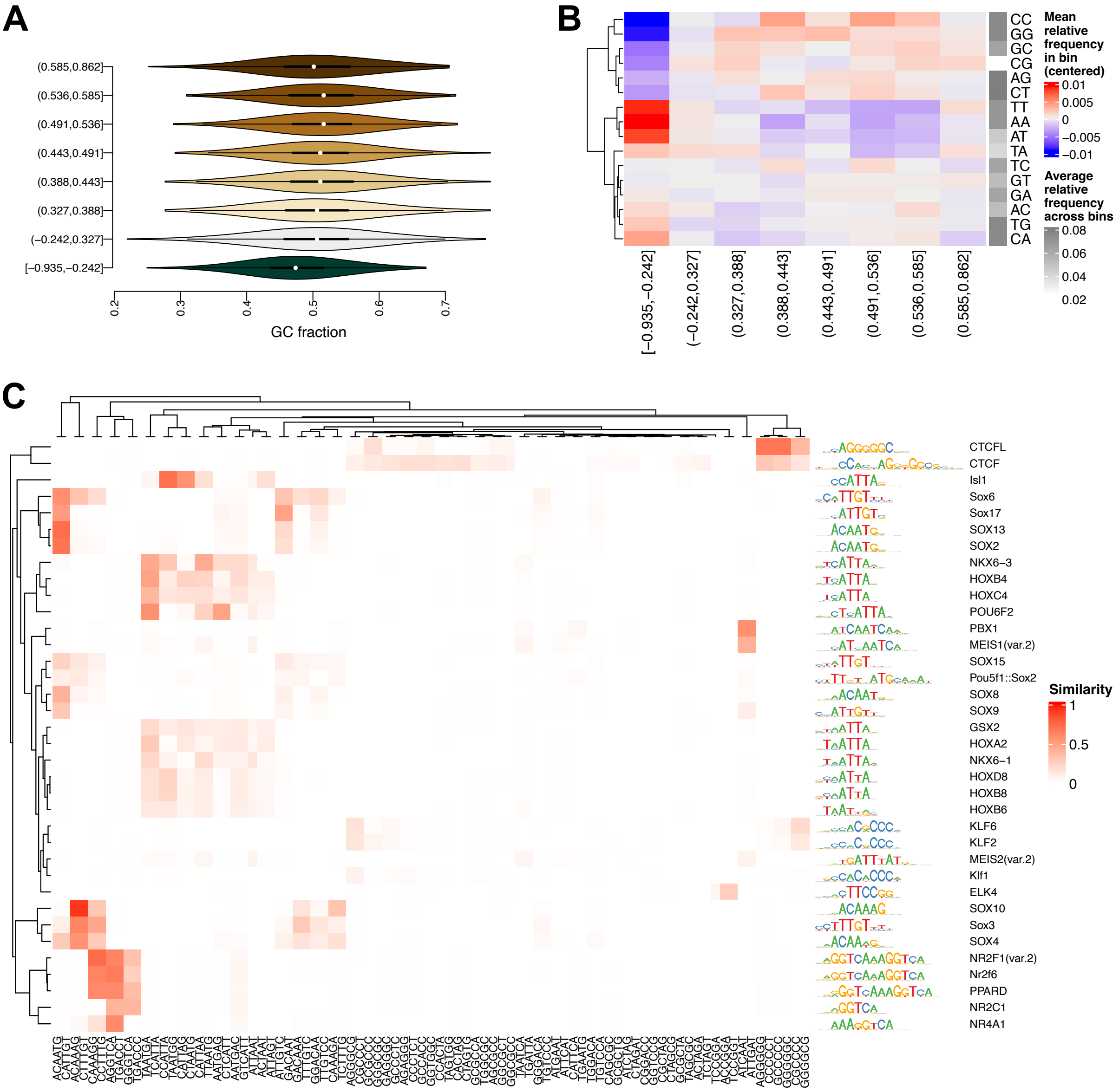

Supplementary Figure 1. Binned enrichment analysis of methylation changes between embryonic stem cells and neuronal progenitors. **A** Distributions of the fraction of G+C bases for sequences in each bin. **B** Differences of dinucleotide frequencies for sequences in each bin, relative to the mean over all bins. **C** Similarities between enriched motifs (rows) and k-mers (columns). Potential gaps in the motif analysis can be identified as k-mers without similar motifs.

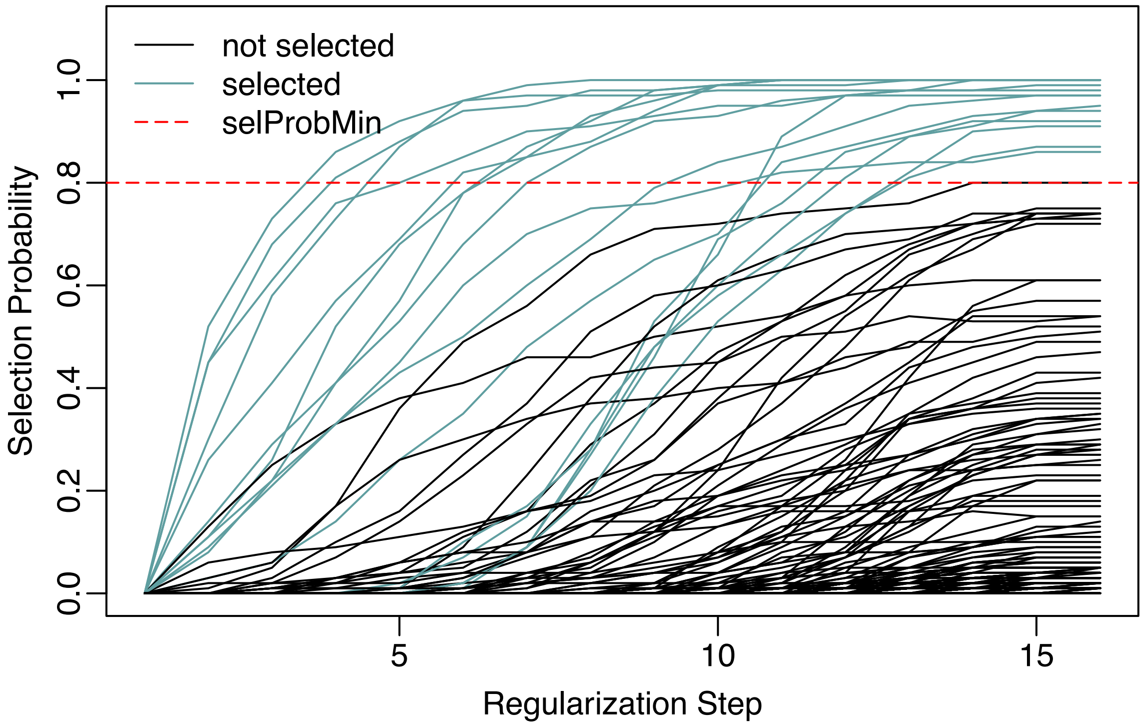

Supplementary Figure 2. Regression-based analysis of accessibility changes between liver and lung. Stability paths showing the selection probability for each motif as a function of the regularization step, with the legend indicating the colors of selected motifs and the selection probability cutoff.
